# Targeting SARS-CoV-2 main protease: a comprehensive approach using advanced virtual screening, molecular dynamics, and in vitro validation

**DOI:** 10.1186/s12985-024-02607-4

**Published:** 2024-12-21

**Authors:** Smbat Gevorgyan, Hamlet Khachatryan, Anastasiya Shavina, Sajjad Gharaghani, Hovakim Zakaryan

**Affiliations:** 1 Laboratory of Antiviral Drug Discovery. Institute of Molecular Biology of National Academy of Sciences, 0014 Yerevan, Armenia; 2 Denovo Sciences Inc, 0060 Yerevan, Armenia; 3 Laboratory of Bioinformatics and Drug Design (LBD), Institute of Biochemistry and Biophysics, University of Tehran, Tehran, Iran

## Abstract

The COVID-19 pandemic, driven by the SARS-CoV-2 virus, necessitates the development of effective therapeutics. The main protease of the virus, Mpro, is a key target due to its crucial role in viral replication. Our study presents a novel approach combining ligand-based pharmacophore modeling with structure-based advanced virtual screening to identify potential inhibitors of Mpro. We screened around 200 million compounds using this integrated methodology, resulting in a shortlist of promising compounds. These were further scrutinized through molecular dynamics simulations, revealing their interaction dynamics with Mpro. Subsequent in vitro assays using the Mpro enzyme identified two compounds exhibiting significant micromolar inhibitory activity. These findings provide valuable scaffolds for the development of advanced therapeutics targeting Mpro. The comprehensive nature of our approach, spanning computational predictions to experimental validations, offers a robust pathway for rapid and efficient identification of potential drug candidates against COVID-19.

**Supplementary Information:**

The online version contains supplementary material available at 10.1186/s12985-024-02607-4.

## Introduction

The COVID-19 pandemic caused by severe acute respiratory syndrome coronavirus 2 (SARS-CoV-2) has had a tremendous global impact, both health-related and socioeconomic [1]. It has already resulted in the deaths of over 7 million people [2], which is estimated to rise further [3]. Hence, it is no surprise that it attracted global attention to fight against this disease or at least moderate its impact on society to the common flu level. The situation is further complicated by the constant rise of SARS-CoV-2 variants, mainly induced by the spike protein mutations that impact the pandemic's clinical and epidemiological character [4]. Several vaccines based on different distinct technology platforms have been developed worldwide. However, none of them provides complete protection against COVID-19, and there is accumulating evidence that the immunological effects triggered by the vaccines diminish over time [5].

In addition to vaccines, several drug candidates are in different stages of preclinical trials [6, 7]. However, at the time of the current article's writing, only three drugs have been approved by the US Food and Drug Administration (FDA). Those drugs are Paxlovid (nirmatrelvir and ritonavir), Veklury (remdesivir), and Lagevrio (molnupiravir). In addition, a handful of drugs have been approved for Emergency Use Authorization. Those are drugs for which, under specified regulatory parameters, the FDA may grant exemptions, enabling the administration of investigational new drugs or off-label applications of approved therapeutics [8, 9]. However, neither drug fully protects from COVID-19, and, in addition, they have serious side effects [10, 11]. Therefore, identifying new effective COVID-19 drug candidates is of paramount importance.

One of the most promising therapeutic targets of SARS-CoV-2 is the main protease, also known as 3CLpro or Mpro [12]. The Mpro is an essential protease in the virus's life cycle and mediates viral transcription and replication. It cleaves the overlapping pp1ab and pp1a polyproteins into functional proteins, a crucial step during viral replication [13]. Among the significant advantages of Mpro as a target is that the human proteases are sufficiently dissimilar and are not targeted by the same drug. On the other hand, the sequences and structures of the Mpro are similar to those found in other beta-coronaviruses, which expedites the drug discovery process by leveraging previously investigated drug candidates [14]. Furthermore, the Mpro is one of the most abundant proteins on the viral surface and is believed to play an essential role in the coronavirus assembly [15]. The crystal structures of Mpro show that it functions as an active homodimer with approximate C2 symmetry and is similar to other coronavirus main proteases. The substrate-binding site of Mpro has a catalytic dyad formed by C145 and H41 amino acids situated at the center of a gap in the substrate-binding site in addition to a conserved water molecule that makes a hydrogen bond with H41 [16].

There is a growing interest in finding new ligands targeting the Mpro of SARS-CoV-2 using in silico simulations. Several studies have utilized generic and custom-made libraries for virtual screening to identify the most promising hits. Once identified, potential compounds are computationally evaluated based on their absorption, distribution, metabolism, excretion–toxicity (ADME-Tox), and drug-likeness profiles. This computational assessment helps streamline the selection of compounds for subsequent in vitro and in vivo evaluation, thereby reducing the time and resources required in traditional drug discovery methods [17, 18].

Virtual screening has been particularly instrumental in the search for novel inhibitors of the Mpro. High-throughput screenings of extensive compound libraries, including FDA-approved drugs and traditional medicines, have identified several candidates with strong binding affinities. These candidates are further validated through molecular docking and dynamics simulations, underscoring the effectiveness of virtual screening in rapidly discovering antiviral agents against COVID-19 [19–21].

Nevertheless, virtual screening in drug discovery faces significant drawbacks, including the frequent occurrence of false positives and false negatives due to the limitations in accurately predicting binding affinities and poses of ligands, which can mislead the identification of potential drug candidates [22]. Additionally, there are challenges in accounting for target flexibility, the presence of metal ions, and active-site water molecules, which can significantly impact the reliability and success rate of virtual screening campaigns [23].

To address these problems, our research team has previously developed an innovative virtual screening approach named Advanced Virtual Screening (AVS) that combines consensus- and ensemble-based docking strategies to achieve higher precision. Compared to other traditional methods, this approach enhances the ranking power and hit rates of structure-based virtual screening [24].

In this study, we integrated ligand-based pharmacophore modeling and structure-based AVS to identify novel scaffolds for COVID-19 therapeutics. Our efficient hybrid approach led to the discovery of 43 potential compounds. Following molecular dynamics simulations and in vitro testing, two compounds demonstrated micromolar inhibitory activity against SARS-CoV-2's Mpro, marking them as candidates for next-step optimizations.

## Materials and methods

### Pharmacophore construction, validation, and screening

#### Ligands collection

ChEMBL (https://www.ebi.ac.uk/chembl/) and BindingDB (https://www.bindingdb.org/) databases have been screened against all ligands with the Mpro target and have biochemical activity. The selected compounds were filtered by their IC_50_ value being less than 20 μM and having no more than two violations of Lipinski's rule of five. The filtered ligands from both databases have been cleaned from duplicates, totaling 155 final filtered ligands.

#### Ligands clustering

The ligands were clustered based on topological fingerprints. The Tanimoto distance between these fingerprints was used with a threshold of 0.7 using the Python RDKit package, a widely used library of cheminformatics tools. Further, the biggest cluster was chosen to construct the pharmacophore model.

#### Pharmacophore model construction

The pharmacophore model was constructed using Ligandscout [25] (v.4.4) software. Two clusters of screened molecules were selected based on the lowest median IC_50_ value and the highest molecule count. Subsequently, 10 different pharmacophore models were generated for each, with the highest scoring the most validated ones based on evaluation selected for further investigation. The 3D structures of ligands were imported and aligned, and protonation states were assigned. Consequently, the pharmacophore models were generated using shared features such as hydrogen bond acceptors/donors, hydrophobic regions, and aromatic rings. Lastly, the pharmacophore models were validated through screening. Decoys were generated based on the active molecules using the DUD.E program [26]. Based on the screening results, the pharmacophore models produced from the biggest cluster and the cluster with the lowest median IC_50_ were chosen for further analysis.

#### Pharmacophore-based screening of the library

The constructed pharmacophore model was imported to the Pharmit online server [27] for the in silico screening of compound libraries. Pharmit is an advanced web-based platform consolidating a suite of computational tools tailored for ligand-based and structure-based drug design methodologies. Notably, it encompasses an extensive array of databases, some containing hundreds of millions of chemical entities, facilitating thorough virtual screenings [28]. The “Exclusive shape: spheres” option was activated, and the following libraries have been screened: CHEMBL30, ChemDIv, Chemspace, MCULE_ultimate, MolPort, and ZINC. Subsequently, the filtered ligands were cleaned from duplicates, and the molecules that violated more than two of Lipinski's rule of 5 were also removed. In the end, 168 unique ligands remained.

### Molecular docking

#### Structure preparation

Eight crystallographic structures of the SARS-CoV-2 Mpro protease were obtained from the Protein Data Bank (PDB) [29] following the criteria of selection of the holo forms, high root-mean-square deviation (RMSD), and structural diversity. The PDB IDs used for this study were 7VLP, 7TE0, 7RFS, 7T43, 7DPU, 7DDC, 6WNP, and 5RHE [30–36]. The protein structures were visualized and analyzed using PyMOL (Schrödinger, LLC). The obtained crystallographic structures were preprocessed by removing water molecules, ions, and any other non-protein entities present in the structures. The bound ligands were removed from each structure, leaving only the protein's coordinates. The resulted cleaned protein structures were subsequently used for docking studies.

#### ICM Pro

The ICM-Pro (v3.9–3) [37] is a robust software package for molecular modeling, drug discovery, and bioinformatics that provides a direct link to the Protein Data Bank (PDB) and enables biologists and chemists to perform complex molecular simulations and analyses. It uses the Monte Carlo simulation method and does a global energy minimization of ligands' internal coordinates in the protein binding pocket. ICM-Pro provides two scoring functions: the "ICM score" and the Potential of Mean Force score (PMF). For each of the eight crystallographic structures, we performed molecular docking analysis. The docking grid map was defined based on the neighbor amino acids interacting with the eight reference ligands extracted from the obtained structures. The size and position of the rectangular grid box were adjusted automatically around the binding site. The box was centered at the co-crystallized molecule and extended 4 Å in any direction. The number of generated conformers for each ligand was 10, and the docking effort was set to 10. For ranking ligands by minimum energy, we used the ICM scoring function, which is based on several ligand–protein interactions: van der Waals energy, hydrophobic free energy gain, hydrogen bonds, and also the internal force field of the ligand. The best conformation out of 10 was picked for each ligand and presented in the final hit list. The binding box coordinates are presented in Table S1.

#### Autodock Vina

AutoDock Vina is a widely used molecular docking software that employs the iterated local search global optimizer to generate docking poses [38]. This optimizer is similar to those used in ICM and the Broyden-Fletcher-Goldfarb-Shanno (BFGS) quasi-Newton method for local optimization. The software utilizes a hybrid scoring function that combines empirical and knowledge-based approaches [39]. In a typical docking procedure, the "exhaustiveness" parameter was set to 8, and standard parameters recommended by the developers were employed. The receptors and ligands were prepared using Autodock Tools. The 3D structures of molecules were formatted into .pdbqt format, which included hydrogen atoms and partial charges. Next, the grid box was defined around the target areas of each protein where ligands were expected to bond. The output by Autodock Vina included nine different poses ranked based on their binding energies. The coordinates for the binding box are detailed in Table S1.

#### Ensemble docking

Ensemble docking was conducted using the eight crystallographic structures of the Mpro to represent the multiple conformations. Ligands were docked into each structure and ranked based on their docking scores, from 1 (best) to 168 (worst). The ranks were summed across all structures to obtain an overall ensemble score for each ligand. Ligands with the lowest ensemble scores were selected for further analysis.

#### Consensus docking

Consensus docking was performed by integrating the results from two docking programs: AutoDock Vina and ICM Pro. These programs utilize different methodologies for calculating their scoring functions. AutoDock Vina employs a scoring function based on a combination of empirical and knowledge-based potentials, focusing on factors such as hydrogen bonding, hydrophobic interactions, and entropy contributions. In contrast, ICM Pro uses an energy-based scoring function that incorporates terms for van der Waals interactions, electrostatic interactions, and solvation effects. Due to these differing approaches, the energy values produced by AutoDock Vina and ICM Pro are not directly comparable. For each program, the 168 compounds were docked against the eight target structures as described in the ensemble docking section. Each compound received a rank for each protein structure within each program based on its docking score. To determine the final consensus rank for each compound, the ranks from both programs and all protein structures were summed, resulting in a total rank score. This rank summation approach was chosen over normalization and averaging to accommodate the distinct scoring scales and methodologies of the two docking programs. By focusing on rank positions rather than raw scores, we ensured that the integration of results from AutoDock Vina and ICM Pro was both meaningful and robust. Compounds with the lowest total rank scores were identified as consistently showing favorable predicted binding affinities across both docking methods and multiple protein conformations.

### MM/PBSA

The Molecular Mechanics-Poisson Boltzmann Surface Area (MM/PBSA) energies were calculated for molecules selected after the consensus docking based on the 20 frames acquired from short (0.02 ns) molecular dynamics (MD) simulations performed on the AutoDock Vina minimum energy pose. A short MD simulation and MM/PBSA calculations were done using the Uni-GBSA open-source tool. It is a comprehensive workflow designed to execute MM/G(P)SA calculations automatically. This workflow encompasses a range of functions, such as preparing topologies, optimizing structures, computing binding free energies, and conducting parameter scans for MM/G(P)SA calculations. Additionally, Uni-GBSA features a batch mode, which permits the simultaneous assessment of thousands of molecules against a single protein target, making it suitable for virtual screening applications [40]. As a configuration file for MD simulations and MM/PBSA calculations, the default.ini configuration file was used with its default parameters, pb calculation mode, and modified parameters for MD simulations (nsteps = 10,000, nframe = 20). Parameters for MD simulations were chosen based on a benchmark study published by Wang et al. [41, 42].

### Molecular dynamics simulations

MD simulations were done using the Uni-GBSA tool. Amber03 and gaff2 force fields were utilized for the receptor and ligands, respectively, to provide accurate intermolecular interactions and conformational sampling. The periodic boundary condition with a cuboid box type was applied to mimic the infinite nature of the system and minimize edge effects. A minimum distance of 10 Å between the solute and the box boundary was maintained to prevent unwanted interactions. The complexes were solvated using the TIP3P water model. Sodium (Na +) and chloride (Cl-) ions were added at a 150 mM concentration to mimic physiological conditions and maintain the system's electro-neutrality. A Monte Carlo barostat with a reference pressure of 1 bar was employed to maintain constant pressure during the simulations. A Langevin thermostat with a collision frequency of 2 ps^−1 was used to regulate the temperature at 309.75 K, representing the initial stages of a viral disease before fever onset. The Particle Mesh Ewald (PME) method with an electrostatic interactions cutoff of 10 Å was utilized for long-range interactions. Hydrogen bonds were constrained using the SHAKE algorithm with a 2 fs integration step. The simulation protocol involved a two-step process: 1 ns of system equilibration followed by 50 ns or 500 ns of conventional MD. This allowed the system to reach a stable, equilibrated state before the production runs, providing a reliable starting point for analyzing the molecular interactions and conformational changes occurring during the simulations. The MD frames of the most active compounds (ID11 and ID35, identified via later experiments) have been clusterized using the gmx cluster program, using the gromos method and putting the cutoff at 0.2 nm. The 2D interaction map of the biggest cluster's average (RMSD deviation) pose has been generated using the ICM pro software.

### Calculation of ADME-Tox parameters

The molecular weight (MW), topological polar surface area (TPSA), partition coefficient (LogP), the number of rotatable bonds, the number of hydrogen bond acceptors (HBA), and hydrogen bond donors (HBD) of selected compounds were calculated using Swiss-ADME web tool [43]. The human intestinal penetration (HIA), blood–brain barrier penetration (BBB-P), P-glycoprotein (Pgp) substrate possibility, and cytochrome P450 3A4 subtype (CYP3A4) inhibition of the compounds were calculated by using the ADMETlab 3.0 web tool [44].

### In Vitro IC_50_ evaluation

WuXi AppTec supplied test compounds 1–14, while 15–16 were purchased from Ambinter. PF-07321332, used as a positive control, was also obtained from WuXi AppTec. These compounds were prepared as 20 mM stock solutions in 100% DMSO and stored at −20℃. SARS-CoV-2_WT Mpro proteins were cloned, expressed in E. coli, and purified by WuXi AppTec (Catalog No: RP200225A, Gene ID: QIZ13716.1). Genscript synthesized the substrate, Dabcyl-KTSAVLQSGFRKM-Glu (Edans). The assay buffer contained 20 mM Tris–HCl (pH 7.3), 100 mM NaCl, 1 mM EDTA, 5 mM TCEP, and 0.1% BSA. The primary instruments used were the Liquid Handler (Labcyte Echo655), Microplate Reader (Molecular Devices SpectraMax M2e), and Incubator (Binder BD-115). Compounds at defined concentrations were serially diluted tenfold with 100% DMSO for 5 doses. They were added to 384-well assay plates using an Echo655 liquid handler. PF-07321332 served as the reference compound. For 100% inhibition controls (HPE), 1 μM of PF-07321332 was used. For no inhibition controls (ZPE), DMSO was added. 25 μL of SARS-CoV-2 Mpro protein, diluted with an assay buffer, was added to assay plates containing compounds using a Multidrop dispenser. The final concentration of Mpro was 25 nM. Compounds and Mpro protein were pre-incubated at room temperature for 30 min. Next, 5 μL of the substrate, diluted with assay buffer, was added to the assay plates using Multidrop. The final volume was 30 μL per well, with a final substrate concentration of 25 μM. Each activity testing point had a background control to normalize fluorescence interference. The final DMSO concentration in the test was 1%. After a 60-min incubation at 30 °C, fluorescence signals (RFU) were detected using a SpectraMax M2e microplate reader at Ex/Em = 340 nm/490 nm. IC_50_ values were calculated using GraphPad Prism software via the nonlinear regression model of log (inhibitor) vs. response (variable slope, four parameters).

## Results and discussion

### Pharmacophore model construction, validation, and screening

#### Ligands collection and clustering

To construct ligand-based pharmacophore models, we obtained inhibitors of the Mpro from the ChEMBL and BindingDB databases, both of which are well-known repositories of biochemical activity data. We selected inhibitors with IC_50_ values below 20 μM and no more than two violations of Lipinski's rule of five, ensuring the selection of potent and drug-like compounds. This filtering process yielded a final set of 155 compounds deemed to be highly reliable inhibitors of the Mpro target. These compounds were subsequently clustered using the Tanimoto coefficient with a similarity threshold of 0.7. The clustering analysis distributed the 155 compounds into 94 clusters, with 64 single-molecule clusters. The largest cluster comprised 11 molecules, exhibiting a mean internal similarity value of 0.757, calculated based on the Tanimoto distance matrix.

#### Pharmacophore model construction

The pharmacophore hypothesis is a fundamental concept in drug discovery, delineating a molecule's essential structural characteristics and spatial orientation that are crucial for its biological or pharmacological activity. This hypothesis serves as a foundation for designing novel drug candidates, enabling researchers to predict and emulate the interactions between a target and a ligand, thereby facilitating the development of potential therapeutics [45].

**Fig. 1:**
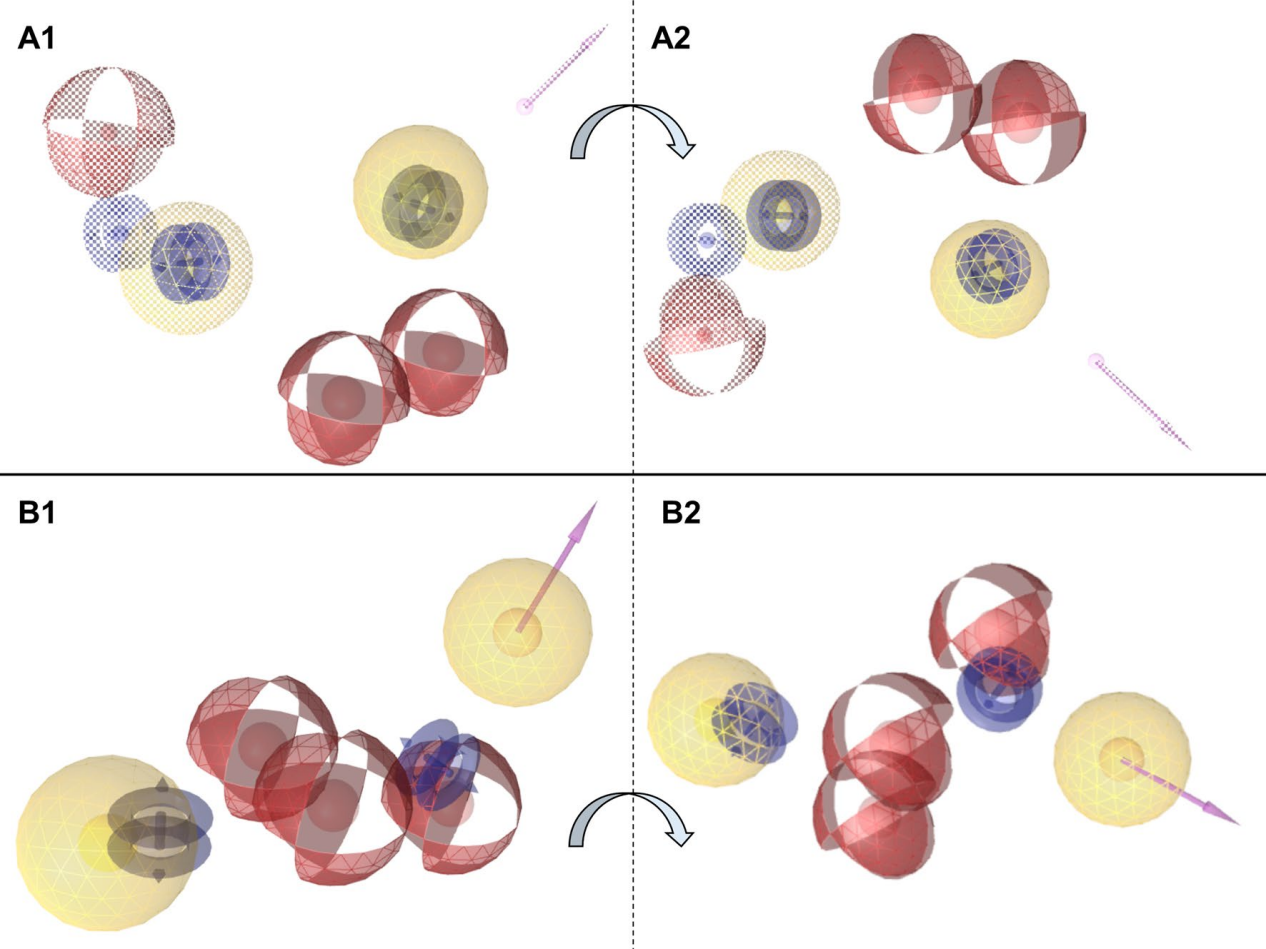
3D pharmacophore models shown in two perspectives. **A1, A2**: pharmacophore model constructed from the largest cluster. **B1, B2**: pharmacophore model constructed from the lowest median IC_50_ cluster. Purple arrows are hydrogen bond donors, red spheres are hydrogen bond acceptors, yellow spheres are hydrophobic groups, and double blue circles are aromatic rings

Several pharmacophore models were constructed and evaluated based on the clusters described above for selectivity and specificity. The two most promising models were built from the largest cluster and the cluster with the lowest median IC_50_ value (61.5 nM). The pharmacophore model from the largest cluster comprised nine features (Fig. 1A1, A2), namely one hydrogen bond donor, three hydrogen bond acceptors, three aromatic rings, and two hydrophobic groups. Whereas the model derived from the cluster with the lowest median IC_50_ included eight features, namely one hydrogen bond donor, three hydrogen bond acceptors, two aromatic rings, and two hydrophobic groups (Fig. 1B1, B2). These models were designed to identify the critical features necessary for ligand binding. Among the two primary models, the first, based on the largest cluster, exhibited superior selectivity (0.169 compared to 0.084 for the second model) and specificity (0.991 compared to 0.984 for the second model), making it the more effective model for virtual screening applications.

#### Pharmacophore-based screening of library

To filter potential hits against the constructed pharmacophore model, we conducted virtual screening against several publicly available chemical libraries, namely CHEMBL30, ChemDIv, Chemspace, MCULE_ultimate, MolPort, and ZINC. The selected pharmacophore model was imported to the Pharmit server, where each library was screened using the default parameters.

The large-scale screening was carried out across six databases on the Pharmit webserver. As presented in Table 1, the total number of compounds within databases ranged from 1,456,000 (ChemDiv) to 126,471,000 (MCULE_ultimate). The total conformers generated spanned from 21,562,000 in ChemDiv to 378,880,000 in MCULE_ultimate. Of the six libraries, MolPort displayed the highest number of hits identified, totaling 385, while ChemDiv showed the least with only 5 hits. Interestingly, although MCULE_ultimate had the largest number of compounds and conformers, it did not have the highest number of hits. Instead, it ranked fourth with 31 hits.

**Table 1:** Screened databases on Pharmit webserver

Library	Total compounds	Total conformers	Hits identified
CHEMBL30	2,186,000	28,970,000	48
ChemDiv	1,456,000	21,562,000	5
ChemSpace	50,181,000	250,205,000	35
MCULE_ultimate	126,471,000	378,880,000	31
MolPort	5,140,000	71,480,000	385
ZINC	13,128,000	122,277,000	286
Total	198,562,000	873,374,000	790

Following the virtual screening process, 790 matches were found. Any duplicate ligands identified in this process were subsequently removed. Moreover, molecules infringing more than two of Lipinski’s rule of 5 were excluded, too. Upon completion of this process, a final count of 168 unique ligands remained.

### Molecular docking analysis

Docking-based virtual screening has been extensively applied in the search for SARS-CoV-2 Mpro inhibitors, aiming to identify promising candidates from vast compound libraries. However, the predictive accuracy is often compromised by weak correlations between docking scores and experimental bioactivity, as well as challenges in distinguishing active from inactive compounds. To enhance the reliability of virtual screening for Mpro inhibitors, rigorous validation protocols, the use of diverse experimental datasets, and the integration of complementary computational methods are essential [46]. Hence, our study implemented advanced virtual screening (AVS), combining ensemble docking using eight different crystallographic structures and implementing two class-leading docking programs: Autodock Vina and ICM Pro. As previously demonstrated, the AVS enhances the trustworthiness of the virtual screening outcomes by employing pre-verified information, collective docking methods, and a consensus approach [24]. This methodology can augment the efficacy of the virtual screening process, especially in the initial identification of potential hit compounds, a critical aspect of real-world screening applications. The results from the two programs were used to calculate the consensus docking score.

#### Ensemble docking

The first step of the AVS method was ensemble docking. This computational technique uses multiple conformations of a target protein to predict the binding geometry and affinity of candidate ligands. Ensemble docking can account for the receptor flexibility and diversity often neglected in conventional docking methods that use a single static protein structure [46]. It has been shown to improve the accuracy and reliability of pose and affinity prediction in various drug discovery applications. Ensemble docking can also enhance the early enrichment power of the models and reduce the computational cost and false positive pose predictions by selecting the most relevant conformations of the target protein [47].

The following crystallographic structures of Mpro were used for ensemble docking: PDB ID: 7VLP, 7TE0, 7RFS, 7T43, 7DPU, 7DDC, 6WNP, and 5RHE. The selection of proteins was based on a clusterization of 342 MPro structures and representative selection from each cluster to include conformational diversity of the binding pocket's interacting amino acids to improve the ensemble docking performance [48]. To ensure the reliability and accuracy of our docking protocols and parameters, we conducted redocking experiments prior to the primary docking analysis. Native ligands were extracted from the eight crystallographic structures and subsequently redocked using both ICM-Pro and AutoDock Vina software. The RMSD between the native ligand poses and the best-scoring docking poses was calculated to assess the performance of each docking program (Table S2). ICM-Pro yielded a mean RMSD of 1.11 Å, with individual RMSD values ranging from 0.62 Å for the structure 7RFS to 2.59 Å for 7DDC. In comparison, AutoDock Vina produced a mean RMSD of 1.28 Å, with the lowest RMSD of 0.62 Å observed for 5RHE and the highest RMSD of 2.62 Å for 7T43. These results demonstrate that both docking programs successfully reproduced ligand poses that closely resemble their native conformations. The consistent performance across multiple structures validates the robustness of our docking protocols and supports the subsequent docking analyses conducted in this study.

A ranking-based scoring system was employed to evaluate the docking performance of each ligand across the ensemble of crystallographic structures. For each structure, ligands were ranked based on their docking scores, with the highest-scoring ligand assigned a rank of 1 and the lowest-scoring ligand assigned a rank of 168. The ranks for each ligand were then summed across all eight structures, resulting in an overall ensemble score for each ligand. Ligands with lower ensemble scores were considered better performers, as they consistently achieved higher docking scores across the panel of crystallographic structures (Table S3).

#### Consensus docking

In the consensus-scoring approach, scores for each compound obtained from the individual software for the same representative protein structure were combined using different docking methods. Consensus docking involves running multiple docking simulations using different algorithms and scoring functions and combining the results to obtain a consensus prediction. By leveraging the strengths of different docking methods, consensus docking aims to improve the accuracy and reliability of binding mode predictions, thereby aiding in the design of new drugs [49, 50].

For this study, consensus docking was performed using the ensemble docking results obtained from Autodock Vina and ICM Pro. Since these docking programs utilize distinct scoring functions, score normalization was required prior to combining the results. Each compound was ranked based on its docking scores in each software. By integrating the individual rankings from Autodock Vina and ICM Pro, we established a comprehensive and more reliable ranking of the compounds. Specifically, after performing ensemble docking on the eight structures, the docking results from the two programs were combined using consensus scoring. The best docking scores for each compound from both programs were averaged to calculate an overall score, and the molecules were then ranked based on this average score (Table S4). As demonstrated in the table, the docking results for the 168 ligands from Autodock Vina and ICM Pro were filtered based on their standardized consensus scores (with lower scores indicating better binding affinity). Consequently, the top ligands with superior consensus rankings were selected for further analysis.

### MM/PBSA

Quantifying binding free energy is a central concern in assessing ligand-receptor binding affinities. The MM/PBSA method balances computational resource requirements and prediction accuracy. It is one of the most widely adopted approaches in virtual screening applications, as corroborated by previous studies [40]. Based on the consensus docking score, the top 43 compounds were analyzed for binding free energy using MM/PBSA (Table S5). The 7RFS structure was chosen for the analysis based on our previous study, where it consistently demonstrated one of the best performances in virtual screening, both in consensus and ensemble docking [48]. The free energy scores ranged from −5.67 kcal/mol (worst) to −18.61 kcal/mol (best), with a mean value of −10.8 kcal/mol. Given the experimental analysis results, 30 compounds with the best results (mean value: −12.16 kcal/mol) were selected for more in-depth molecular dynamics simulation studies.

### Molecular Dynamics

Molecular dynamics (MD) is a computational method widely employed in drug discovery to simulate the temporal evolution of atomic and molecular interactions at an atomistic level. It provides detailed insights into the dynamic behavior of biomolecules, including their conformational flexibility, interaction profiles, and thermodynamic properties, enabling a deeper understanding of the molecular mechanisms underlying biological processes and drug-target interactions [51]. MD simulations play a crucial role in understanding the mechanisms of drug-target interactions and predicting the binding affinity of small molecules to target proteins. By simulating the dynamic behavior of a drug molecule within the target protein's active site, valuable information can be gained about how drugs bind, their stability, and the conformational changes that occur upon binding [52].

After performing the MD analysis on the 30 compounds screened by MM/PBSA, the protein–ligand complexes were examined, and the most stable complexes were selected for further analysis (Fig S1–S5). Based on the evidence in the literature [53], we assumed that the stability of complexes, specifically those maintaining stability for a minimum duration of 50 ns, may serve as a reliable indicator of their functional performance in a biological context. Ligands, when bound and retained within the protein's active site for an adequate length of time, could effectively influence and modify the protein's functionality. Accordingly, 16 compounds were selected for in vitro analysis. These compounds demonstrated the highest stability within the protein's active site, maintaining stable interactions for at least 50 ns based on RMSD analysis of the ligands against the protein. This selection criterion ensured that only the most promising candidates, based on their predicted functional performance in a biological context, were advanced for experimental validation.

### ADME-Tox prediction

Understanding the absorption, distribution, metabolism, excretion, and toxicity (ADME-Tox) properties is fundamental to drug design and development, as these properties shape therapeutic compounds' pharmacokinetics, efficacy, and potential side effects [54]. Therefore, in addition to our MD analysis, we computationally evaluated the ADME-Tox profiles of the top 16 compounds using the SwissADME and ADMETlab 3.0 online tools. Specifically, we analyzed various physicochemical and biological attributes, including molecular weight (MW), topological polar surface area (TPSA), partition coefficient (LogP), number of rotatable bonds (RB), hydrogen bond acceptors (HBA), and hydrogen bond donors (HBD). These properties, along with the compounds' SMILES annotations and molecular structures, are presented in Table 2. Furthermore, we calculated human intestinal absorption (HIA), blood–brain barrier penetration (BBB-P), P-glycoprotein (P-gp) substrate potential, and cytochrome P450 3A4 (CYP3A4) inhibition profiles (Table S6).

**Table 2:** ADME properties of top 16 compounds

ID	SMILES	MW	TPSA (Å^2^)	LogP	RB	HBA	HBD	
0	O = c1[nH]c2ccc(Nc3nc4nonc4nc3N/N = C/c3cc4c(cc3[N +](= O)[O-])OCO4)cc2[nH]1	476.36	214.05	1.2	6	11	4	
2	C[C@@H](Nc1ccc(C(= O)Nc2ccc3ccccc3c2)cc1[N +](= O)[O-])c1ccc2[nH]c(= O)[nH]c2c1	467.48	135.6	3.62	7	4	4	
4	Oc1ccc2c(c1)oc1c(-c3ccc(O)c(O)c3)c3c(O)nn(C4CCCC4)c3nc12	417.41	124.77	3.47	2	7	4	
11	Oc1ccc2c(c1)oc1c(-c3ccc(O)c(O)c3)c3c(O)nn(C4CCCCC4)c3nc12	431.44	124.77	3.76	2	7	4	
14	Oc1ccc(-c2c3oc4c(O)c(O)ccc4c3nc3c2c(O)nn3C2CCCCCC2)cc1O	461.47	145	3.78	2	8	5	
17	Oc1ccc(-c2c3oc4cc(O)ccc4c3nc3c2c(O)nn3C2CCCC2)cc1	401.41	104.54	3.84	2	6	3	
18	Oc1ccc(-c2c3oc4cc(O)ccc4c3nc3c2c(O)nn3C2CCCCCC2)cc1	429.47	104.54	4.43	2	6	3	
20	Cc1c(O)ccc2c1oc1c(-c3ccc(O)c(O)c3)c3c(O)nn(C4CCCCC4)c3nc12	445.47	124.77	4.16	2	7	4	
22	O = C1C[C@@H](c2cccc3cccnc23)c2c(cc(O)c3c(= O)c(O)c(-c4ccc(O)c(O)c4)oc23)O1	483.43	150.32	3.14	2	9	4	
26	Cc1[nH]c2ccnn2c(= O)c1CC(= O)Nc1ccc2[nH]nc(C(N) = O)c2c1	365.35	151.03	0.67	5	5	4	
29	Oc1ccc(-c2c3oc4c(O)c(O)ccc4c3nc3c2c(O)nn3C2CCCCCCC2)cc1	459.49	124.77	4.44	2	7	4	
30	Cc1c(O)ccc2c1oc1c(-c3ccc(O)cc3)c3c(O)nn(C4CCCCCCC4)c3nc12	457.52	104.54	5.1	2	6	3	
31	COc1ccc2cc(S(= O)(= O)NN(Cc3cccc(C(= N)N)c3)C(= O)N3CCC(C)CC3)ccc2c1	509.62	137.2	2.86	9	6	3	
32	COc1cc(-c2c3oc4cc(O)ccc4c3nc3c2c(O)nn3C2CCCCC2)cc(O)c1O	461.47	134	3.84	3	8	4	
35	Oc1ccc(-c2c3oc4c(O)c(O)ccc4c3nc3c2c(O)nn3C2CCCCC2)cc1Cl	465.89	124.77	4.32	2	7	4	
41	COc1cc(-c2c3oc4cc(O)ccc4c3nc3c2c(O)nn3C2CCCCCCC2)cc(O)c1O	489.52	134	4.44	3	8	4	

*MW* molecular weight, *TPSA* topological polar surface area, *LogP* partition coefficient, *RB* number of rotatable bonds, *HBA* hydrogen bond acceptors, *HBD* hydrogen bond donors

The results demonstrate that most of the compounds exhibit characteristics typical of drug-like molecules, such as appropriate molecular weights, an optimal balance of lipophilicity and polarity, and suitable numbers of hydrogen bond donors and acceptors. Additionally, these compounds showed no calculated toxicity and good biological membrane penetration. The notable exception is compound ID31, which was predicted to inhibit CYP3A4, has only moderate intestinal absorption, and is a non-substrate for P-gp. Overall, these findings suggest that at least 15 of the top 16 compounds have a high potential to exhibit favorable pharmacokinetics and bioavailability, making them promising candidates for further drug development and testing.

### In Vitro IC_50_ evaluation

Computational screening provides valuable insights into compounds' potential interactions and efficacy. Yet, it is essential to validate these predictions through in vitro experiments to ensure accuracy and reliability. In vitro tests offer tangible evidence of a compounds' activity and possible side effects. This critical step substantiates computational findings and further refines the selection process before advancing to costly and time-consuming in vivo studies or clinical trials [55].

We evaluated the inhibitory activity of the 16 small molecule compounds against the Mpro using a SARS-2 Mpro enzyme assay. The assay was performed with 25 nM of SARS-2 Mpro and a substrate concentration of 25 μM. The tested compounds were measured for their half-maximal inhibitory concentration (IC_50_) values, which indicate the concentration of the compound required to inhibit 50% of the Mpro activity.

**Fig. 2:**
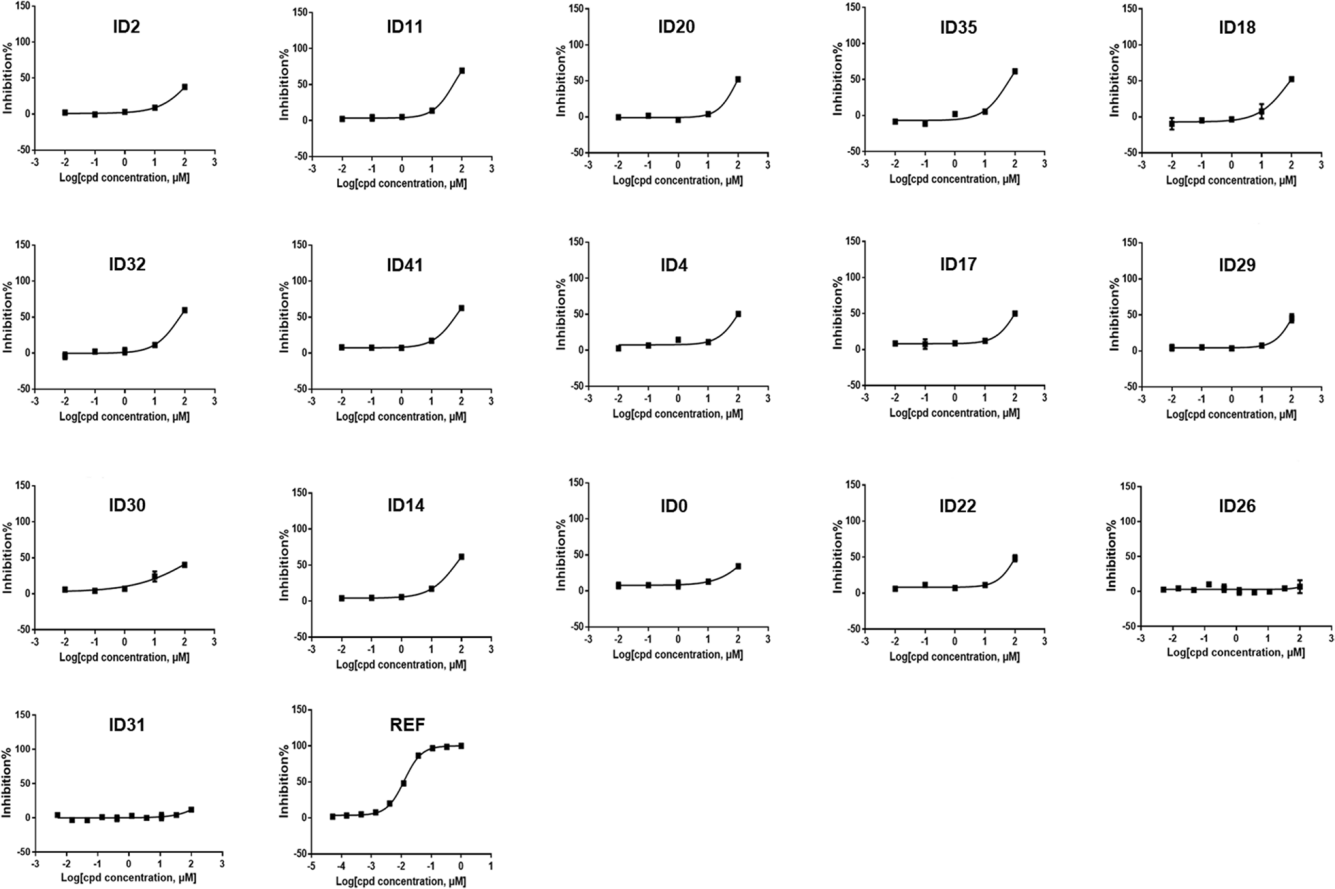
In Vitro IC_50_ Evaluation of top 16 compounds. REF—Reference

Seven of the 16 compounds tested (ID11, ID20, ID35, ID18, ID32, ID41, and ID14) exhibited IC_50_ values below 100 μM (Fig. 2), suggesting a potential inhibitory effect on Mpro activity. Out of them, the most potent compounds were ID11 and ID35, with IC_50_ values of 54.39 μM and 59.39 μM, correspondingly. The remaining nine compounds demonstrated IC_50_ values greater than 100 μM, indicating limited or no inhibitory effects on Mpro activity. Although our compounds are considerably less potent than the reference (0.013 μM), they may still hold promise for further optimization and development as potential antiviral agents targeting SARS-CoV-2 Mpro. Nevertheless, due to financial constraints, only the 16 out of 30 compounds were subjected to in vitro testing; therefore, we invite other researchers to evaluate the remaining 14 compounds to further explore their potential as Mpro inhibitors.

### Long molecular dynamics analysis of ID11 and ID35

Long MD simulations, particularly those over extended timescales like 200 ns or more, are vital in drug discovery for understanding protein-drug and protein–ligand interactions. These extended simulations are crucial for capturing the full range of protein motions and conformational changes, providing a comprehensive view of how drugs interact with protein targets. Shorter simulations may miss important conformations and dynamics, leading to less accurate drug design [56].

**Fig. 3:**
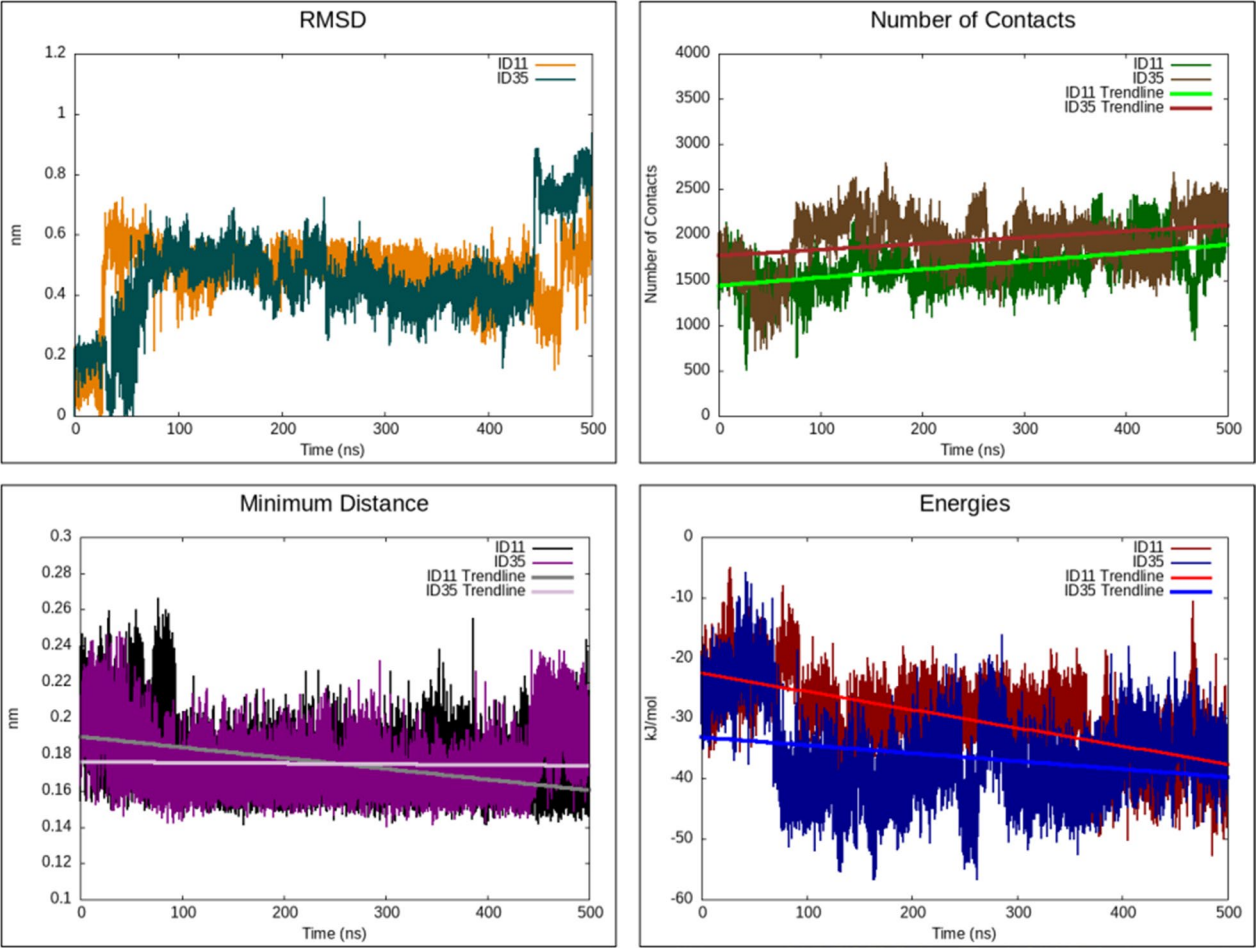
The 500 ns MD analysis of ID11 and ID35. RMSD: Root-mean-square deviation of ID11 and ID35. Number of Contacts: Number of contacts between the ligand and the protein. Minimum Distance: Minimum distance between the ligand and the protein. Energies: Interaction energy profiles of ID11 and ID35

Accordingly, long MD simulations of 500 ns duration were performed on two compounds with the most promising IC_50_ values (ID11 and ID35). These simulations aimed to understand better the interactions between the selected compounds and the Mpro. Post-MD analyses calculated parameters such as binding energy, minimum atomic distance, and the number of atomic contacts within the protein–ligand complex (Fig. 3). The binding energy of a protein–ligand complex is a critical determinant of the interaction strength. It is typically estimated by subtracting the aggregate energies of the individual, unbound protein, and ligand entities from the total energy of the bound complex. The minimum atomic distance measures the shortest spatial separation between any pair of atoms or atomic groups within the system. For instance, within the context of protein–ligand interactions, this parameter signifies the smallest distance between any atom within the protein and any atom in the ligand. A smaller minimum distance often implies a more potent or direct interaction. The number of contacts, defined as the total count of atom pairs within a specified cutoff distance, measures the interaction interface between the protein and the ligand. In this study, atom pairs (each pair consisting of an atom from the protein and one from the ligand) closer to a predefined cutoff distance of 0.6 nm were considered "in contact." This metric provides insights into the extent of the interface between the protein and the ligand during their interaction since, up to this distance, various bonds can form, including hydrogen bonds, ionic interactions, van der Waals forces, hydrophobic interactions, π-π stacking, and cation-π interactions, which collectively contribute to the specificity and stability of the complex.

As demonstrated in Fig. 3, the analysis of ID11 shows a consistent trend in complex-ligand interaction energy, number of contacts, and minimum distance over the MD simulation. Specifically, the interaction energy decreases, and the number of contacts increases, which indicates strong binding interactions and stability of the complex. Despite some fluctuations in RMSD, the overall pattern suggests that ID11 maintains stable interactions within the active site, aligning well with the in vitro results. For ID35, similar patterns were observed, albeit to a lesser extent. The decrease in interaction energy and increase in the number of contacts are present, though not as pronounced, and the minimum distance remains relatively stable throughout the simulation. Altogether, these observations collectively support the strong interactions between the compounds and Mpro during the simulation, indicating potential stability and efficacy despite the observed RMSD variations. Furthermore, the stabilities of both complexes were confirmed by the visual inspection of the simulation videos, where it is clearly visible that both ID11 and ID35 remain stable in the binding pocket throughout the 500 ns simulations, despite some minor fluctuations (Supp. Videos S1 and S2).

**Fig. 4:**
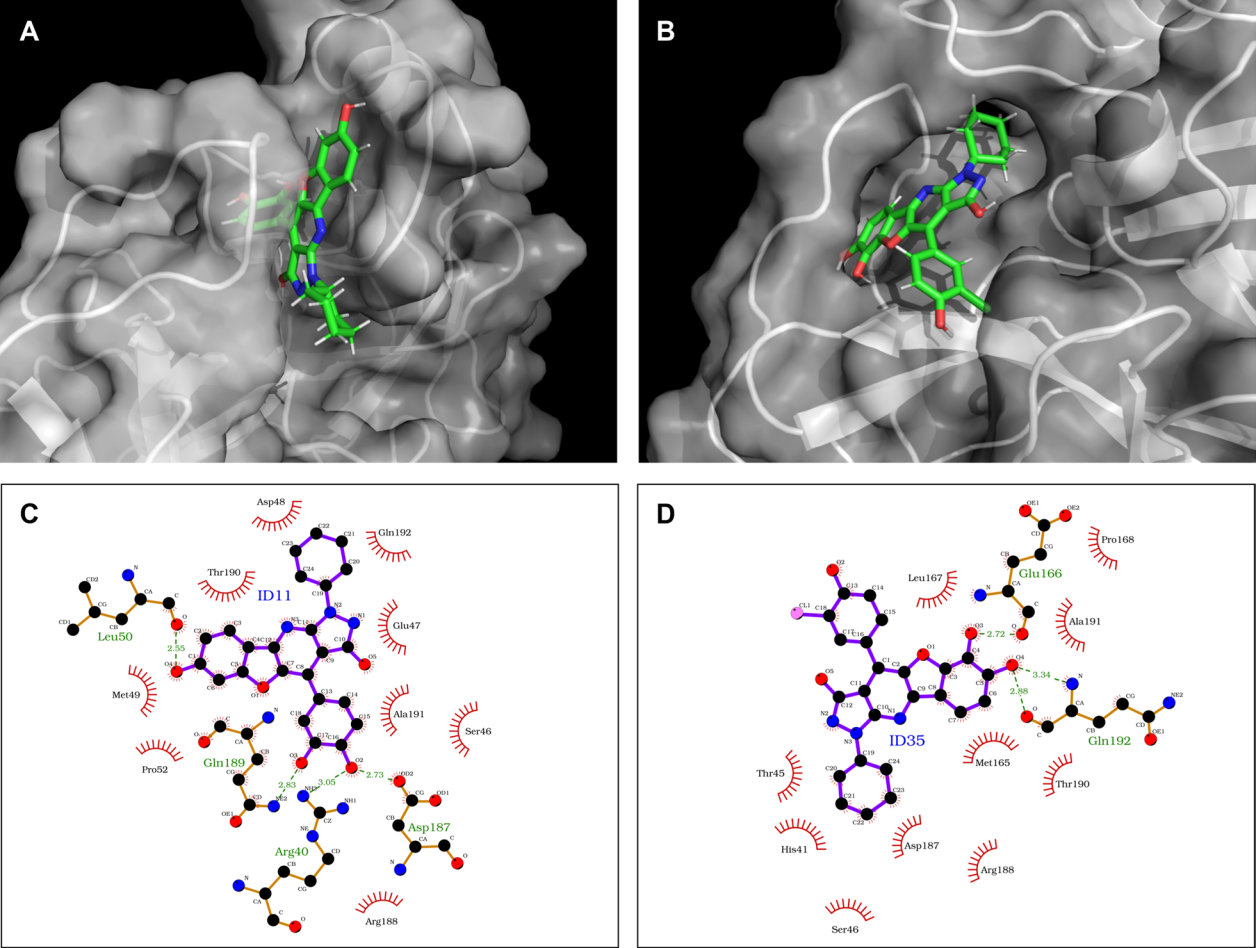
3D representations and 2D interaction maps of the most frequent poses of ID11 and ID35 MD simulations. **A** and **B** the 3D representation of ID11 and ID35 compounds and the Mpro binding pocket, respectively. **C** and **D** 2D interaction maps of ID11 and ID35 with the amino acids of the binding pocket

To comprehensively evaluate the interactions of the two ligands with the binding site amino acids, we conducted a detailed analysis to elucidate the molecular interactions underlying their observed bioactivity. MD trajectories were clustered based on the RMSD values of the conformational frames, resulting in the identification of nine distinct clusters for each ligand. From the largest cluster, the pose with the highest average interaction energy was selected for further examination. This pose was then used to generate a detailed 2D interaction map of the ligands within the binding pocket (Fig. 4), highlighting the key residues involved in binding and their specific interactions.

The interaction analysis revealed that the ligands forms hydrogen bonds with His163 and Asn142, as well as extensive hydrophobic interactions with His163, Asn142, His41, Met49, Phe140, Leu141, Ser144, Cys145, Glu166, and Gln189. These results align with established findings in the literature, particularly regarding the importance of Cys145 and His41, which constitute the catalytic dyad central to the enzymatic activity of Mpro. This detailed interaction profile provides a deeper understanding of the structural basis for ligand binding and its potential contributions to the observed inhibitory activity [16].

The results further confirm our assumption that ID35 and ID11 directly affect the activity of Mpro by interacting with the essential amino acids in the binding pocket. Therefore, these two compounds can serve as important milestones for further optimizations and, subsequently, in finding potent SARS-COV2 medicines.

## Conclusion

This study presents an integrated and comprehensive approach to identifying and characterizing novel inhibitors targeting the SARS-CoV-2 main protease (Mpro). By merging ligand-based pharmacophore modeling with ensemble and consensus docking, followed by detailed molecular dynamics simulations and in vitro assays, we have developed a robust pipeline for drug discovery. The ligand-based pharmacophore model facilitated the identification of 168 unique candidate inhibitors, which were subjected to advanced virtual screening (AVS) techniques. Detailed molecular dynamics (MD) simulations played a crucial role in refining our selection to 16 top-ranking compounds by providing insights into the dynamic behavior and stability of the ligand–protein interactions. Although the IC₅₀ values of even the two most promising compounds indicate moderate potency, their unique structural features and inhibitory activities render them promising candidates for future drug development and novel research. Our findings underscore the efficacy of this integrated computational-experimental approach in accelerating the drug discovery process and providing valuable scaffolds for the development of effective therapeutics against COVID-19. The identified bioactive compounds present strong candidates for further medicinal chemistry efforts and subsequent optimization studies, paving the way for potential clinical applications.

## Supplementary Information

Additional file 1.

Additional file 2.

Additional file 3.

Additional file 4.

Additional file 5.

Additional file 6.

Additional file 7.

Additional file 8.

Additional file 9.

Additional file 10.

Additional file 11.

Additional file 12.

Additional file 13.

## Data Availability

No datasets were generated or analysed during the current study.
