# Supplementary figures and images for: Targeting SARS-CoV-2 main protease: a comprehensive approach using advanced virtual screening, molecular dynamics, and in vitro validation
Source: Virol J. 2024 Dec 21;21:330. doi: 10.1186/s12985-024-02607-4 (PMC11662536; doi:10.1186/s12985-024-02607-4)

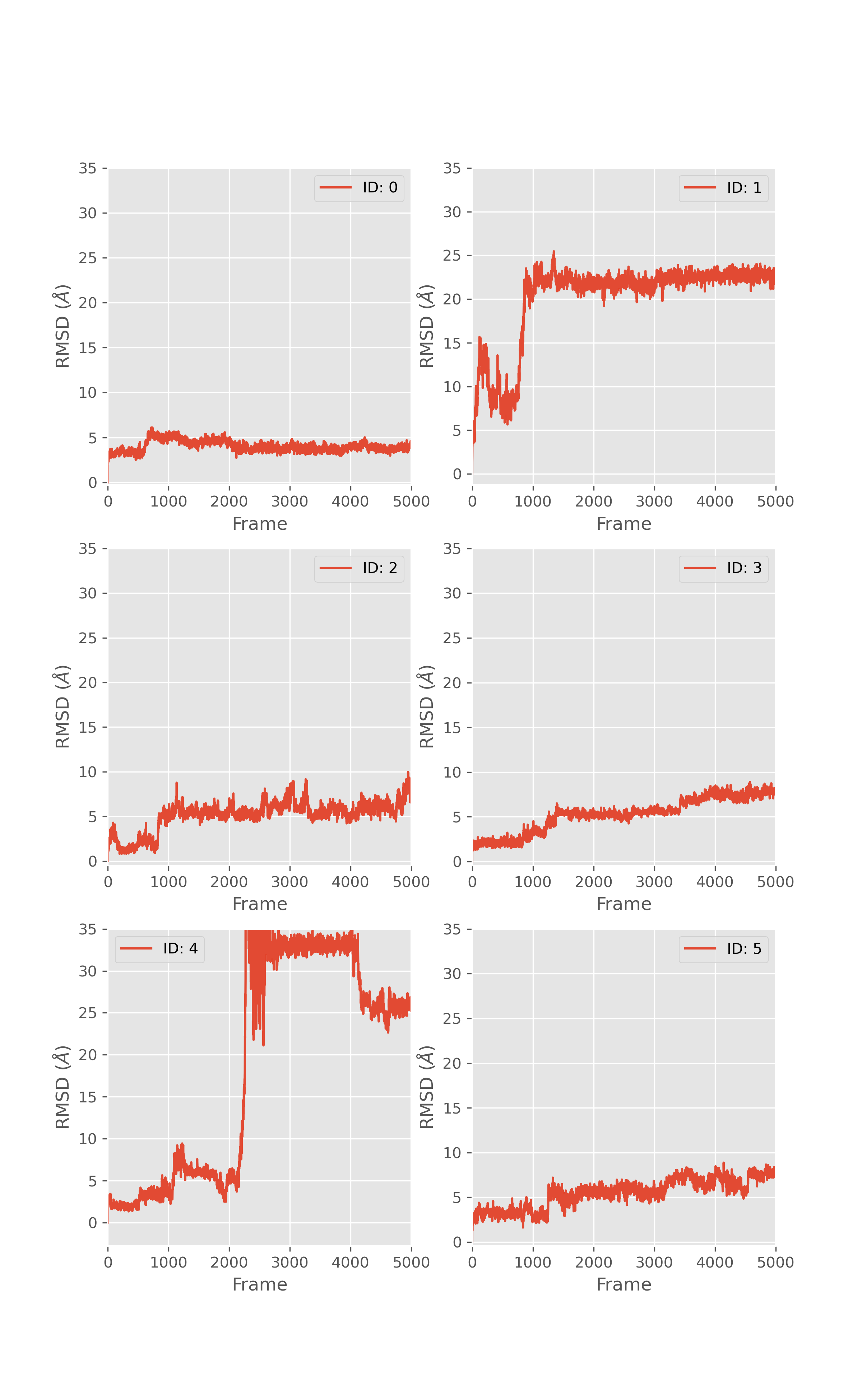

Supplement: Supplementary file 1 — Additional file 1. [file 12985_2024_2607_MOESM1_ESM.png]

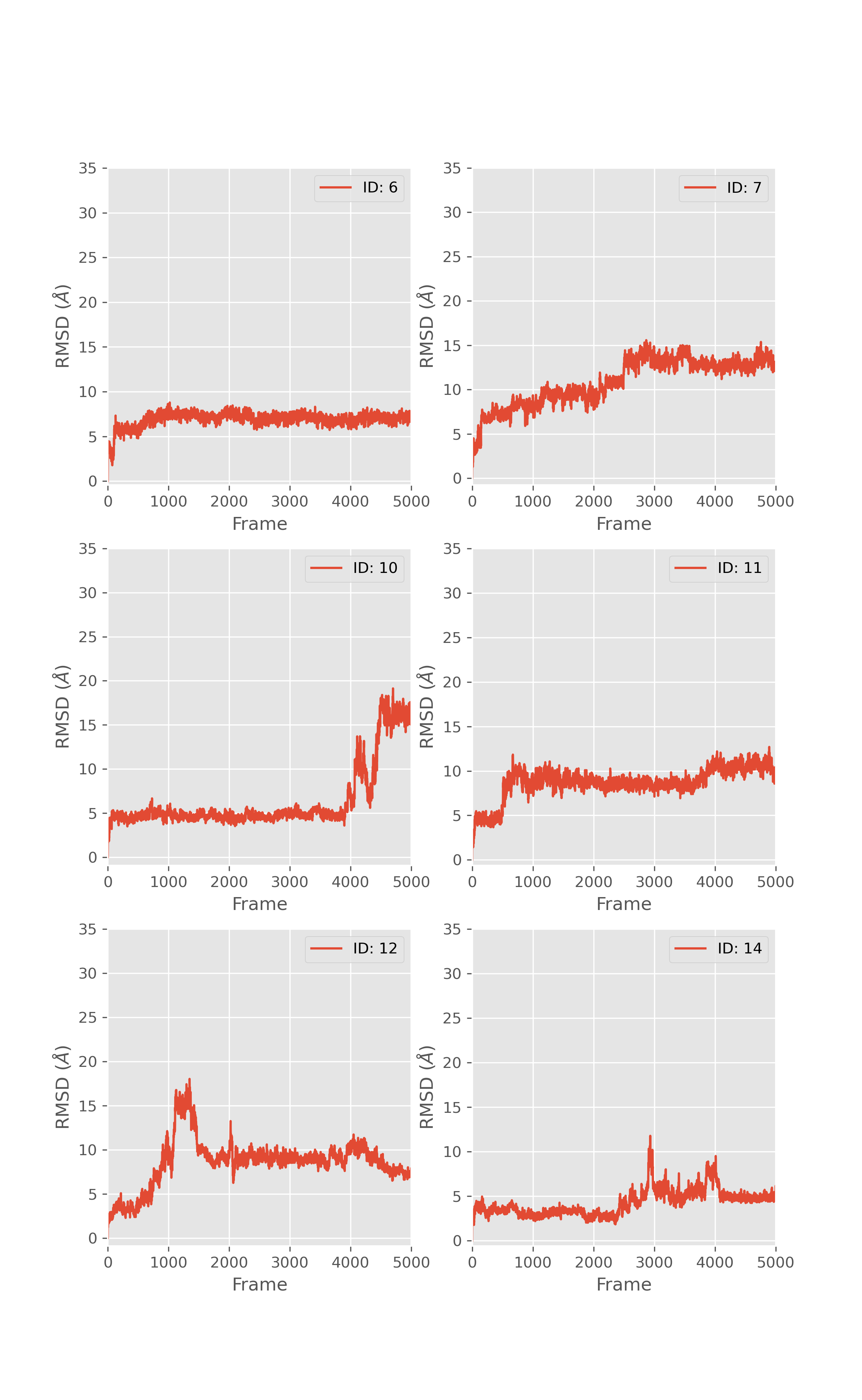

Supplement: Supplementary file 2 — Additional file 2. [file 12985_2024_2607_MOESM2_ESM.png]

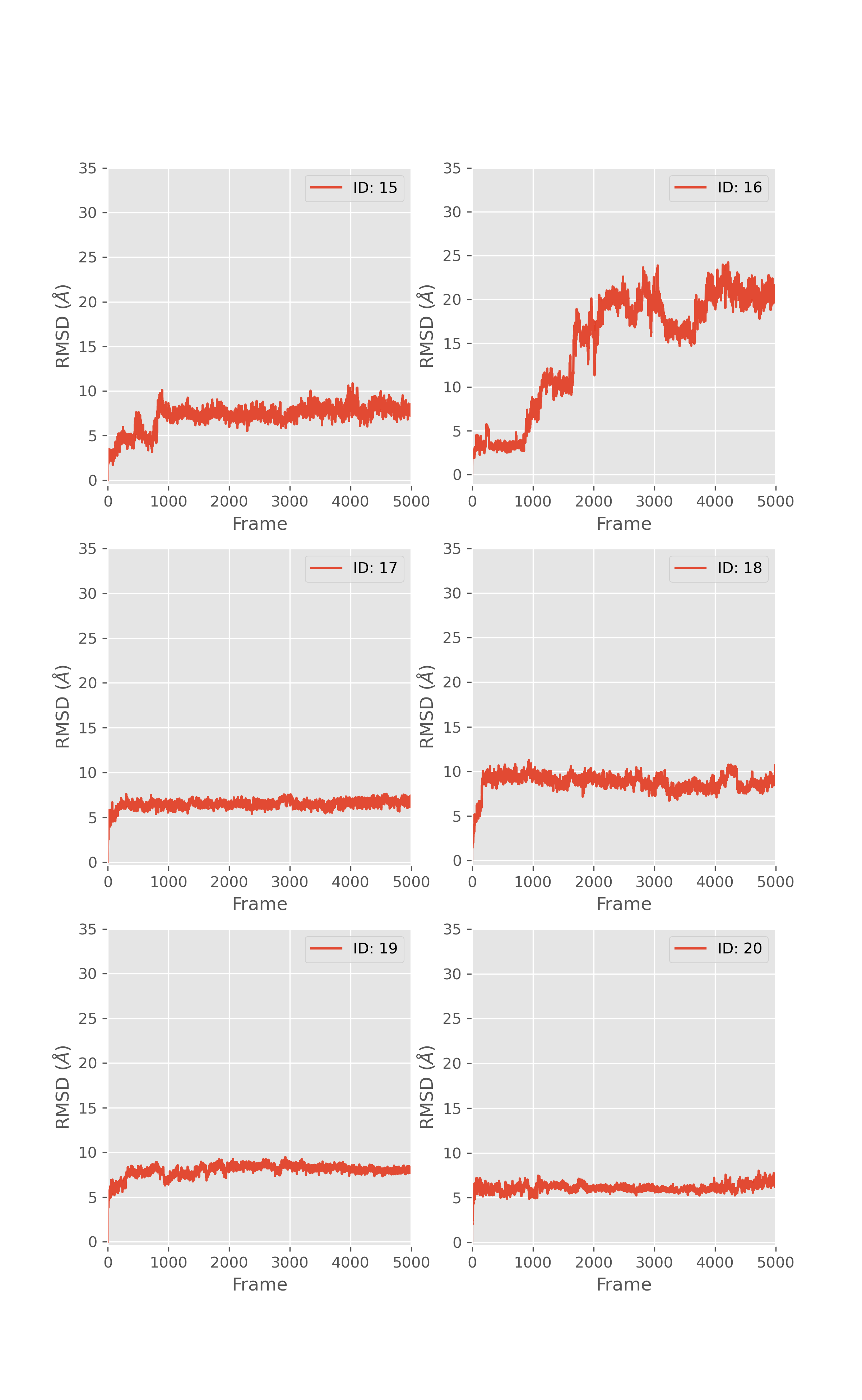

Supplement: Supplementary file 3 — Additional file 3. [file 12985_2024_2607_MOESM3_ESM.png]

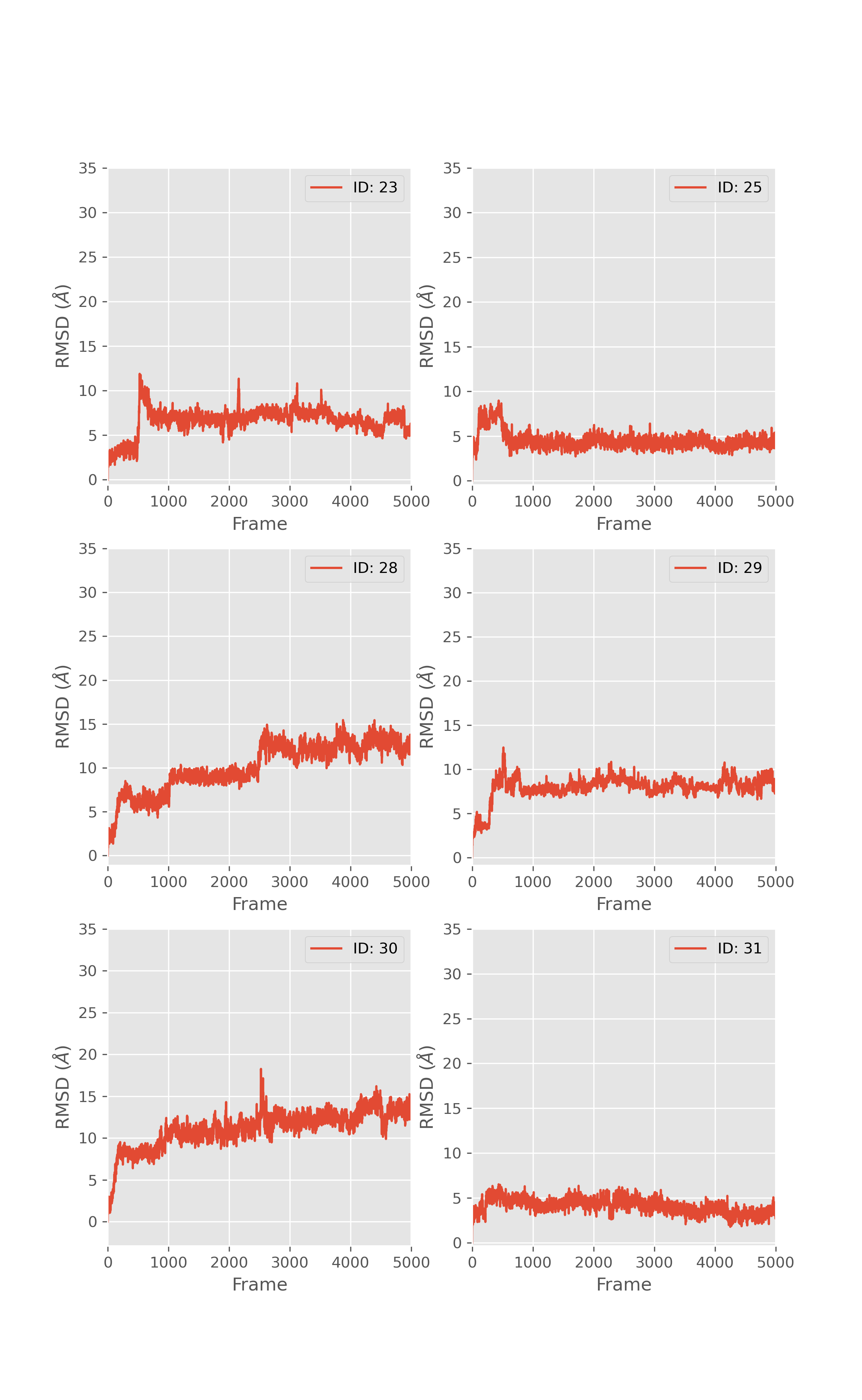

Supplement: Supplementary file 4 — Additional file 4. [file 12985_2024_2607_MOESM4_ESM.png]

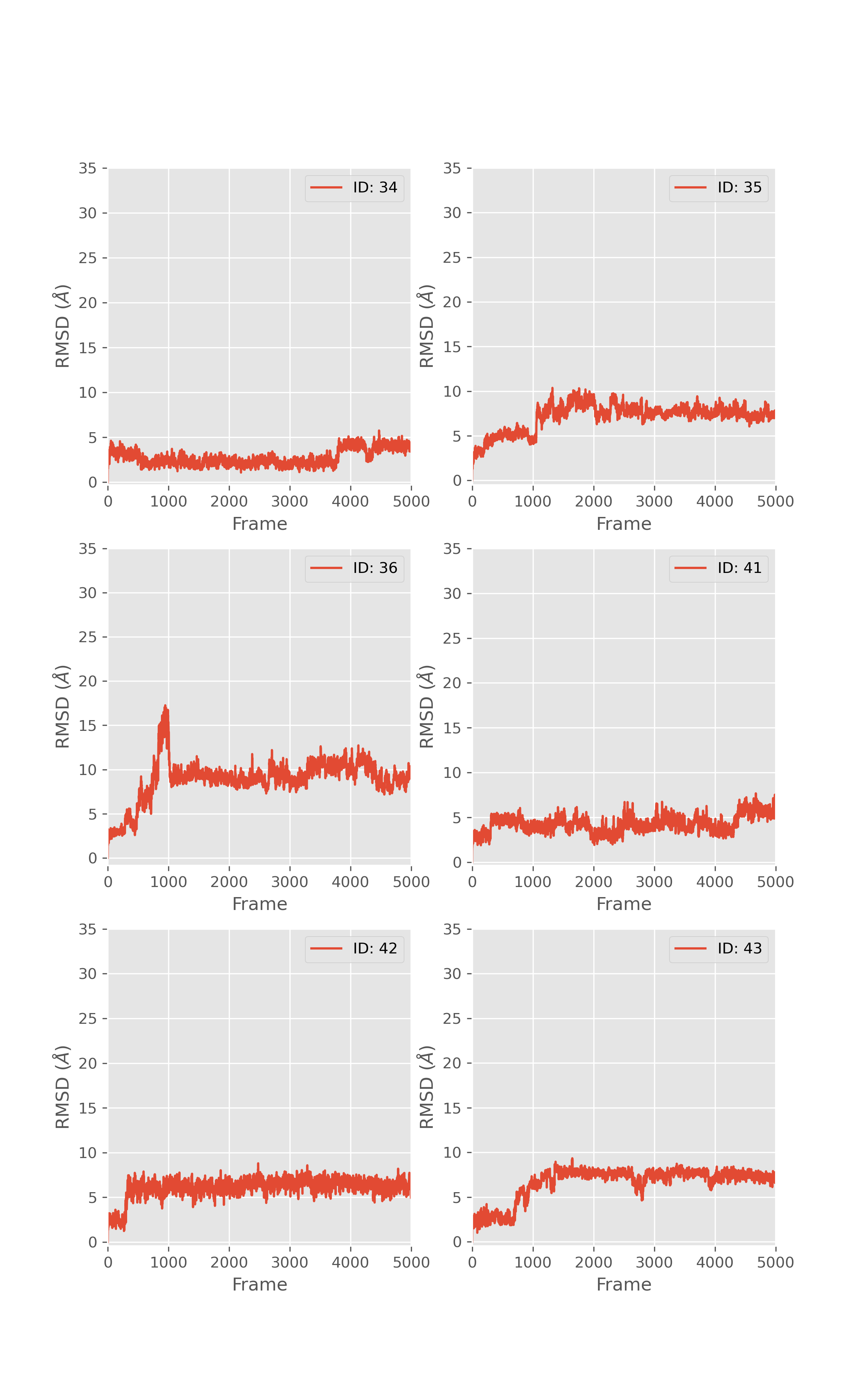

Supplement: Supplementary file 5 — Additional file 5. [file 12985_2024_2607_MOESM5_ESM.png]
